# Infection of Kissing Bugs with *Trypanosoma cruzi*, Tucson, Arizona, USA

**DOI:** 10.3201/eid1603.090648

**Published:** 2010-03

**Authors:** Carolina E. Reisenman, Gena Lawrence, Pablo G. Guerenstein, Teresa Gregory, Ellen Dotson, John G. Hildebrand

**Affiliations:** University of Arizona, Tucson, Arizona, USA (C.E. Reisenman, P.G. Guerenstein, T. Gregory, J.G. Hildebrand); Centers for Disease Control and Prevention, Atlanta, Georgia, USA (G. Lawrence, E. Dotson); 1 Current affiliation: Consejo Nacional de Investigaciones Cientificas y Técnicas, Diamante, Argentina.

**Keywords:** Chagas disease, Trypanosoma cruzi, triatomines, Triatoma, kissing bugs, Arizona, Triatoma rubida, vector-borne infections, zoonoses, research

## Abstract

Triatomine insects (Hemiptera: Reduviidae), commonly known as kissing bugs, are a potential health problem in the southwestern United States as possible vectors of *Trypanosoma cruzi*, the causative agent of Chagas disease. Although this disease has been traditionally restricted to Latin America, a small number of vector-transmitted autochthonous US cases have been reported. Because triatomine bugs and infected mammalian reservoirs are plentiful in southern Arizona, we collected triatomines inside or around human houses in Tucson and analyzed the insects using molecular techniques to determine whether they were infected with *T. cruzi*. We found that 41.5% of collected bugs (n = 164) were infected with *T. cruzi*, and that 63% of the collection sites (n = 22) yielded >1 infected specimens. Although many factors may contribute to the lack of reported cases in Arizona, these results indicate that the risk for infection in this region may be higher than previously thought.

## Abstract 2

A survey of triatomine insects found that 41.5% were infected with the causative agent of Chagas disease.

Chagas disease is endemic throughout Mexico and Central and South America, with ≈7.7 million persons infected, 108.6 million persons considered at risk, 3–3.3 million symptomatic cases, an annual incidence of 42,500 cases (through vectorial transmission), and 21,000 deaths every year (*1*–*3*). This disease is caused by the protozoan parasite *Trypanosoma cruzi*, which is transmitted to humans by blood-sucking insects of the family Reduviidae (Triatominae). Although mainly a vector-borne disease, Chagas disease also can be acquired by humans through blood transfusions and organ transplantation (*2*–*6*), congenitally (from a pregnant woman to her baby) (*7*), and through oral contamination, e.g., foodborne (*8*). Acute infection can be lethal, and cardiomyopathy develops in 25%–30% of infected persons (*1*). Although neither a vaccine against infection nor a completely effective treatment for chronic Chagas disease currently exists (*2*,*9*), treatment is now recommended for acute infections, congenital infections, infections in immunosupressed persons, and infections in children (*10*).

Although historically Chagas disease has been considered restricted to Latin America (*1*,*3*), the disease is becoming a serious health issue in the United States because of the presence of a notable number of blood donors seropositive for *T. cruzi* (*11*–*13*). Notably, a small number of the seropositive blood donors have never left the United States. Only 7 autochthonous cases of this disease have been reported in the United States, all in the southern half of the country (*14*–*19*). The most recent reported case of autochthonous transmission of *T. cruzi* occurred in 2006 near New Orleans, Louisiana (*18*). Many cases of Chagas disease in the United States, however, may be overlooked because the early phase of the infection is often asymptomatic (*9*,*16*), and health professionals are largely unaware of this disease. In Arizona, humans may be at a greater risk for vectorial transmission of the disease than previously thought because human populations are rapidly expanding into habitats where infected triatomines (*20*–*22*) and wild mammalian reservoirs such as packrats, mice, armadillos, raccoons, and opossums (*23*–*27*) are plentiful. Chagas disease is actively transmitted in domestic cycles involving dogs in southern Texas (*20**,**28*), where >50% of triatomines collected inside or near the homes of persons were found to be infected with *T. cruzi* (*19*,*20*). Studies conducted many decades ago found that triatomines in California, Arizona, and New Mexico were also infected with *T. cruzi* (*22*–*25*,*29*).

Arizona is noteworthy as the state with the highest number of triatomine–human contacts reported in the United States (American Association of Poison Control Centers, www.aapcc.org/DNN; Arizona Poison and Drug Information Center, University of Arizona Health Sciences Center, www.pharmacy.arizona.edu/outreach/poison). In southern Arizona, triatomine bugs live in association mostly with the white-throated woodrat (*Neotoma albigula*) (*24*,*26*). Triatomine bugs have wingless nymphal stages and winged adults. During their dispersal season (beginning of May through July), adult insects, attracted by light, reach human habitations (*30*–*32*). *Triatoma rubida* is by far the most common species (Figure 1), but *T. protracta* and *T. recurva* are also found (*30*,*32*). *T. rubida* was associated with a clinical case of Chagas disease in the city of Guaymas, Mexico, although this bug is perhaps a different subspecies than the one found in Arizona (*33*).

**Figure 1:**
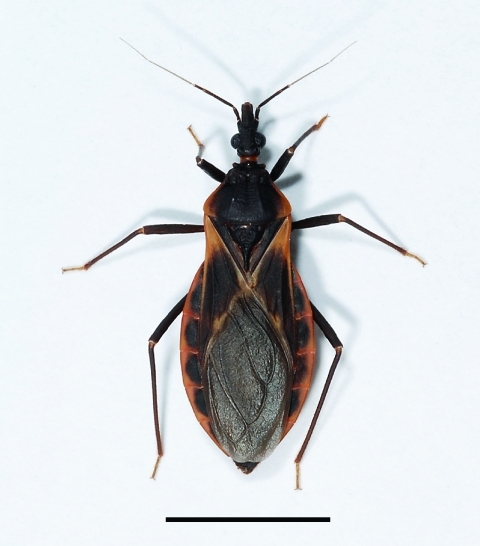
Adult female kissing bug of the species *Triatoma rubida*, the most abundant triatomine species in southern Arizona. Scale bar = 1 cm.  **Photo credit line: Photograph by C. Hedgcock.**

To our knowledge, the most recent comprehensive studies about the infection rates by *T. cruzi* in triatomines from Arizona were conducted >45 years ago (*21*,*22*), by using microscopy to detect the presence of live parasites in the insect’s gut or feces. In 1943, Wood (*22*) found an overall infection rate of 4% in triatomines (28 of 699) from Arizona collected over a 3-year period. In 1964, Bice (*21*) collected triatomines from packrat dens in what is today a densely populated area in metropolitan Tucson, Arizona, and found that 7.5% and 19.5% of *T. rubida* and *T. protracta* bugs, respectively, were infected with *T. cruzi* (*21*). A recent study that used molecular methods, but was based on a small sample, found that 1 in 4 *T. protracta* and 0 of the 20 *T. rubida* bugs examined were infected with *T. cruzi* (*34*).

To estimate the current potential of vectorial transmission of *T. cruzi* disease in southern Arizona, we investigated the infection rate of triatomines collected inside and around houses in metropolitan Tucson (Pima County), Arizona. Tucson is the second largest metropolitan area in Arizona with a population (as of 2007) of 1,003,235, of which 462,103 persons live in areas where triatomines are plentiful (*35*).

## Materials and Methods

### Collection of Insects

Triatomine insects were obtained by issuing public requests asking residents of metropolitan Tucson (32°13′18′′N, 110°55′35′′W), Arizona, to collect bugs found inside or around their houses. Insects that reach houses, as opposed to those directly collected from nests of wild animals, are of greatest epidemiologic importance because they have the highest chance of contact with humans. Collectors were instructed to use a container and not to touch or handle the insects with their bare hands, and they were usually informed about the way that Chagas disease is transmitted. In a preliminary study conducted in 2005, we found that some triatomine bugs were infected with *T. cruzi* (C.E. Reisenman et al., unpub. data). We therefore conducted a more extensive study in 2006. For each bug, we recorded, whenever possible, the collection site (address), insect species, stage, sex (if adults), and date of collection as well as any other information the collector provided. Collected insects were individually placed in 95% ethanol immediately after collection or upon death and stored at 4°C until analysis. Insects were collected during May 15–December 18, 2006.

### Analysis of *T. cruzi*

Each insect was analyzed by PCR for the presence of *T. cruzi*. Before analysis the insect was removed from ethanol and dried overnight in a petri dish to remove traces of ethanol before DNA extraction. The lower abdomen of each bug was detached with a sterile razor blade and homogenized with a ceramic ball, or placed in a 1.5-mL microfuge tube with phosphate-buffered saline (<80 μL) and homogenized with a hand-held mortar.

DNA was extracted following the instructions provided with the QiaAmp DNA Blood Mini Kit (QIAGEN 51106; QIAGEN, Valencia, CA, USA). The DNA was amplified by PCR according to an established *T. cruzi* sample-processing protocol (*36*) by using the *T. cruzi*–specific primers TCZ1 (5′-CGAGCTCTTGCCCACACGGGTGCT-3′) and TCZ2 (5′-CCTCCAAGCAGCGGATAGTTCAGG-3′), which amplify 188 bp of a repetitive nuclear sequence (*15*). For the minicircle locus, DNA was amplified by using primers S35 (5′-AAATAATGTACGGGKGAGATGCATGA-3′) and S36 (5′-GGGTTCGATTGGGGTTGGTGT-3′) (*37*), which amplify a 330-bp minicircle sequence. A 50-μL reaction containing 0.4 μM of each primer, 20–40 ng of template DNA, and DNA polymerase (GoTaq; Promega, Madison, WI, USA, or Platinum Taq; Invitrogen, Carlsbad, CA, USA) was prepared. Primers for PCR were made at the Centers for Disease Control and Prevention (Atlanta, GA, USA) core facility or acquired from Invitrogen. The cycling parameters for the reactions with the TCZ1 and TCZ2 primers were as described (*36*). The cycling parameters for the reactions that used the S35 and S36 primers were an initial denaturation at 95°C for 10 min, 35 cycles of amplification at 95°C (30 s each), 58°C (30 s each) and 72°C (1 min each), and a final extension at 72°C for 10 min. Samples were processed in a Mastercycler Gradient Thermocycler Machine (Eppendorf, Hauppauge, NY, USA) or an iCycler (Bio-Rad, Hercules, CA, USA). PCR products were subjected to electrophoresis on 1.5% agarose gels, stained with ethidium bromide, and visualized by using UV transillumination with AlphaImager program (Alpha Innotech, San Leandro, CA, USA). All PCRs were run with a positive control of known *T. cruzi* DNA and with a negative control in which template DNA was omitted. Results that were positive for both sets of primers were considered positive. If a sample was positive for only 1 set of primers, then the products of the PCR were cloned (pGem-T Easy Vector System; Promega) and sequenced (Big Dye Terminator, v1.1 and ABI 31 30xl Genetic Analyzer; Applied Biosystems, Foster City, CA, USA). Cloned sequences were compared with sequences in GenBank to determine if the amplified sequence belonged to the *T. cruzi* genome. A random sample of ≈15% negative samples (n = 11) was analyzed along with positive samples to exclude the possibility of false-negative samples.

## Results

### Insect Collection and Demographics

A total of 164 triatomine bugs (158 [96.3%] *T. rubida*, 5 [3%] *T. recurva*, and 1 [0.6%] *T. protracta*) were collected by volunteers and analyzed for *T. cruzi*. Most of the collected *T. rubida* were adults (93.6%, n = 151). Of the 141 adult *T. rubida* identified by sex, 87 were females (62%) and 54 were males (38%). The proportion of females to males was statistically different from a 1:1 sex ratio (χ^2^ = 8.2, df = 1, p = 0.004).

Twenty-two collectors provided a total of 142 insects, with each collector contributing a variable number of insects per night (range 1–10, median 2). A single collector provided 73 insects collected on 16 nights throughout the dispersal season. Twenty-two additional bugs were collected by an unknown number of anonymous persons. Information about the specific location where insects were collected was obtained for 84% (n = 139 insects provided by 19 collectors) of the insects. These 139 insects (all *T. rubida*) were obtained from 17 collection sites distributed in 6 of the 8 metropolitan Tucson areas corresponding to the cardinal and ordinal points of the compass, and from 2 collection sites in central Tucson (Table). Because insects were collected by volunteers rather than by using systematic collection methods (i.e., light traps set up in all geographic areas), the information in the Table serves the sole purpose of reporting where insects were collected and does not constitute an estimate of the abundance of insects per area.

**Table:** Collection sites and collected insects per area, triatomine insects survey, metropolitan Tucson, Arizona, USA, 2006*

Area	No. collection sites (% with insects infected with *Trypanosoma cruzi*)	No. insects collected (% infected with *T. cruzi*)
Central	2 (100)	2 (100)
North	1 (0)	2 (0)
Northeast	1 (100)	2 (50)
Northwest	6 (66)	14 (43)
South	0	0
Southeast	3 (66)	11 (45)
Southwest	2 (100)	19 (42)
East	0	0
West	4 (100)	88 (40)

*Information about collection sites was obtained for 139 of the 164 bugs collected. An individual collector from the western area provided an unusually large number of insects (n = 73).

**Figure 2:**
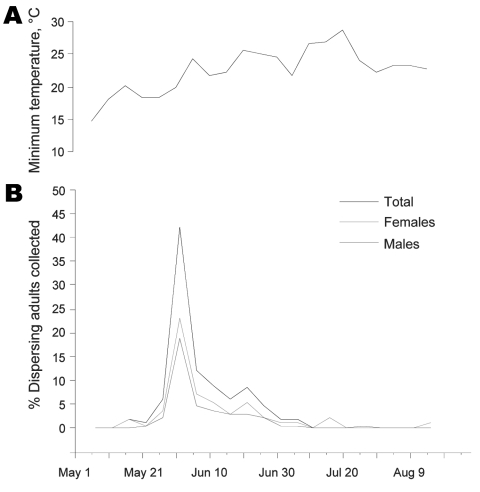
Temporal pattern of adult *Triatoma rubida* insects collected in metropolitan Tucson, Arizona, USA, May–August, 2006. A) Average minimum daily temperature recorded in 2006 during the period shown (data obtained from www.wrh.noaa.gov/twc/climate/reports.php). B) Percentages of all adults (n = 134), males (n = 52), and females (n = 82) collected during the period, in 5-day intervals (e.g., the percentage of insects collected during May 15–19 is represented on May 17). Information about sex or collection date was not available for 16 adults, so they were not included in this plot.

Adult *T. rubida* insects were collected in or around houses from mid-May through the end of August (Figure 2). Most adults were collected in the last days of May and first week of June (Figure 2, panel B); a total of 61% of insects were caught during May 25–June 8. This peak in insect collections coincides with a typical, sustained increase in minimum temperatures that enables insects to fly at night (*32*) (Figure 2, panel A). Bugs were collected steadily throughout the last week of June; only 13 adults (8%) were collected during the rest of the dispersal season, which extends to the end of August. Although insects were not collected by using systematic methods, peak collection periods coincide with the peak dispersals reported by Ekkens (*32*).

### Analysis of Infection by *T. cruzi*

We found that 68 (41.5%) of the 164 bugs collected were infected with *T. cruzi*. Twenty-four (35%) of the samples were positive by both set of primers and therefore were considered positive. The remaining 44 (65%) positive samples were positive for S35/S36 only, but all of them were confirmed positive by cloning and sequencing, thus excluding the possibility of false-positive results. No samples were positive for TCZ1/TCZ2 and negative for S35/S36.

Of the 22 identified sites or houses where insects were collected, 14 (63%) had at least 1 bug infected with *T. cruzi*. Infected bugs were found in 7 of 8 areas, including central Tucson (Table). The percentage of infected bugs per area was variable (median 43%, range 0%–100%), likely due to the low number of bugs (1–2) collected in certain areas (e.g., central, north, northeast). The mean ± SD percentage of infected bugs per area, considering only those areas where >10 insects were collected, was 42.5% ± 1.0% (4 geographic areas, n = 132 insects). Similarly, to estimate the prevalence of infection per collection site, we selected sites where at least 5 bugs were collected. The mean ± SD number of infected bugs per collection site was 47.2% ± 5.7% (n = 7 collection sites in 4 geographic areas, n = 120 insects). This percentage was slightly higher (48.8 ± 6.6%, n = 6 collection sites) when a site where a large number of bugs were collected (n = 73) was excluded from the analysis.

The prevalence of infection by *T. cruzi* among triatomine species was variable, as reported (*21*), although a larger sample is necessary to confirm this prevalence. Forty-one percent of *T. rubida* (n = 158) bugs, 60% of *T. recurva* (n = 5) bugs, and the single *T. protracta* bug collected were infected with *T. cruzi*. Because only a few *T. recurva* and *T. protracta* bugs were collected, we restricted all further analysis to *T. rubida*. Forty-two percent of nymphs (n = 7), 40.1% of females (n = 87), and 40.0% of males (n = 54) of *T. rubida* were found to be infected with *T. cruzi*. Among adults, the probability of infection was independent of sex (χ^2^ = 0.015, df = 1, p>0.9, by χ^2^ contingency analysis). Infected bugs were found throughout the year; the median number of infected insects per 5-day collection period during the dispersion season (mid-May through mid-July) was 27% (range 17%–67%).

## Discussion

To our knowledge, almost no information has been collected during the last half-century on the incidence of infection by *T. cruzi* in triatomine bugs from Arizona (but see below). We found that 41.5% of the 164 collected bugs, most of which were *T. rubida*, were infected with *T. cruzi*, and that 63% of houses or sites where insects were collected had at least 1 specimen infected. Most bugs collected were adults, and this winged life stage is known to be the main driver of dispersal (*38*). Although most bugs were collected inside or around human houses from May through the end of June, infected bugs were collected throughout the period of study. Specimens of the less abundant species *T. recurva* and *T. protracta* were also found to be infected. Samples that were positive with only 1 set of primers were confirmed by sequencing of the amplified DNA, excluding the possibility of false-positive results. In contrast with our results and previous research by others (*21*,*22*), a recent study found that none of the *T. rubida* bugs collected in the Tucson area were infected with *T. cruzi* (*34*). This discrepancy might be explained by the use of a different set of primers, the low numbers of insects examined (n = 20 in the aforementioned study), or bias in the insect sample, such as few collection sites. Furthermore, the infection rate reported here is much higher than that reported in earlier studies in Arizona, which ranged from 4% to 9% (*22*,*24*,*29*). Those studies were conducted by using microscopy that visualized the presence of the parasite in the insect gut; therefore, discrepancies maybe be attributed to differences in the sensitivity of the methods used (e.g., *16*).

The infection rates reported in this study, however, are in line with those reported in other recent systematic studies. For instance, 51% of triatomines (mostly *T. gerstaeckeri*) collected from several areas in Texas were infected (n = 241), with many insects found near human dwellings (*19*). In Guaymas, in northwestern Mexico, 81% of *T. rubida* collected in houses and in the peridomicile (n = 279) were infected with *T. cruzi* (*39*). The fact that in that region adults and juveniles of *T. rubida* were found inside houses indicates a progressive domiciliation of this otherwise wild species, probably related to housing developments in triatomine habitats (*39*). In our study, immature stage (nymphs) insects collected inside houses were also infected, but the numbers are too small to draw any definitive conclusions. If these houses are sites of bug colonization, then the risk for human infection may be higher than in houses where only adult insects were found and removed. Nevertheless, because most immature insects in our study were found 1–4 months after the peak of dispersion (i.e., they are likely the offspring of adults that invade houses earlier) rather than consistently throughout the year, *T. rubida* bugs do not appear to be in the process of becoming domiciliated in Arizona.

Why have there been no reports of autochthonous cases of Chagas disease in Arizona despite our finding that 41.5% of bugs are infected with *T. cruzi*? In southern Arizona, triatomines live in close association with the sylvatic animal reservoirs upon which they feed (*26*) and apparently have a low capacity for domiciliation, although juvenile insects (the offspring of dispersing adults) can be found in houses near beds and readily feed on humans if necessary. Good housing conditions (e.g., lack of crevices in walls or ceilings) do not favor the permanent domiciliation of the insects, but this may not be the case in rural areas where housing materials provide shelter for the insects. Under those circumstances, colonization of human habitats might be favored because at least half of dispersing adults were female and likely gravid (C.E. Reisenman, unpub. data). In principle, the parasite can be transmitted to humans when infected insects that invade houses defecate on the skin of a human host upon feeding. Although a recent study reported that *T. rubida* and *T. protracta* do not defecate while feeding (*34*), our current investigations indicate that this is not the case for *T. rubida* bugs in all stages and for both sexes (C.E. Reisenman, unpub. data). Pet dogs can become infected by contamination with excreta but also by contact with the oral mucosa when they instinctively chew insects that might be infected (*40*).

Other reasons that might explain why Chagas disease is so rare in the United States are the following: misdiagnosis of the early infection (*9*,*16*), low insect vectorial capacity (*34*), or low infectivity of the genetic lineage of the *T. cruzi* parasites present in local insects and mammals, although this remains to be investigated. Bice (*21*) showed the presence of *T. cruzi* parasites in the heart muscle of a mouse inoculated with feces from an adult *T. rubida* bug collected in the Tucson area. Should the lineage of *T. cruzi* present in southern Arizona correspond to that associated with the pathogenic form of Chagas disease, the data presented here suggest that vectorial transmission of the disease in the area is possible.
